# Supplementary material for: Exploring the scale effect of nonpoint source pollution risk on water quality in Lake Basins of Central Yunnan Plateau using the Minimum Cumulative Resistance model
Source: PeerJ. 2024 Oct 18;12:e18247. doi: 10.7717/peerj.18247 (PMC11493029; doi:10.7717/peerj.18247)
Supplement: Supplemental Information 1 [file peerj-12-18247-s001.docx]

Remote sensing image data descriptions (*Landsat8 OLI* ).

Data Source: https://glovis.usgs.gov/

| **Sensor** | **Date** | **GTM** | **Scene ID** | **Spatial resolution (m)** |
| --- | --- | --- | --- | --- |
| OLI | 2018-03-01 | 03:34:41 | LC81290432018060LGN00 | 30 |

Three Lakes Basin Data and Source

| **Data type** | **Year** | **Spatial resolution** | **Data source** |
| --- | --- | --- | --- |
| *Terrain* | *2018* | *30m* | https://glovis.usgs.gov/ |
| *Land use type* |  |  | http://www.resdc.cn |
| vegetation coverage |  |  | http://www.dsac.cn/ |
| Water Quality |  | － | Yuxi Environmental Protection Bureau |
