# Supplementary figures and images for: Exploring the scale effect of nonpoint source pollution risk on water quality in Lake Basins of Central Yunnan Plateau using the Minimum Cumulative Resistance model
Source: PeerJ. 2024 Oct 18;12:e18247. doi: 10.7717/peerj.18247 (PMC11493029; doi:10.7717/peerj.18247)

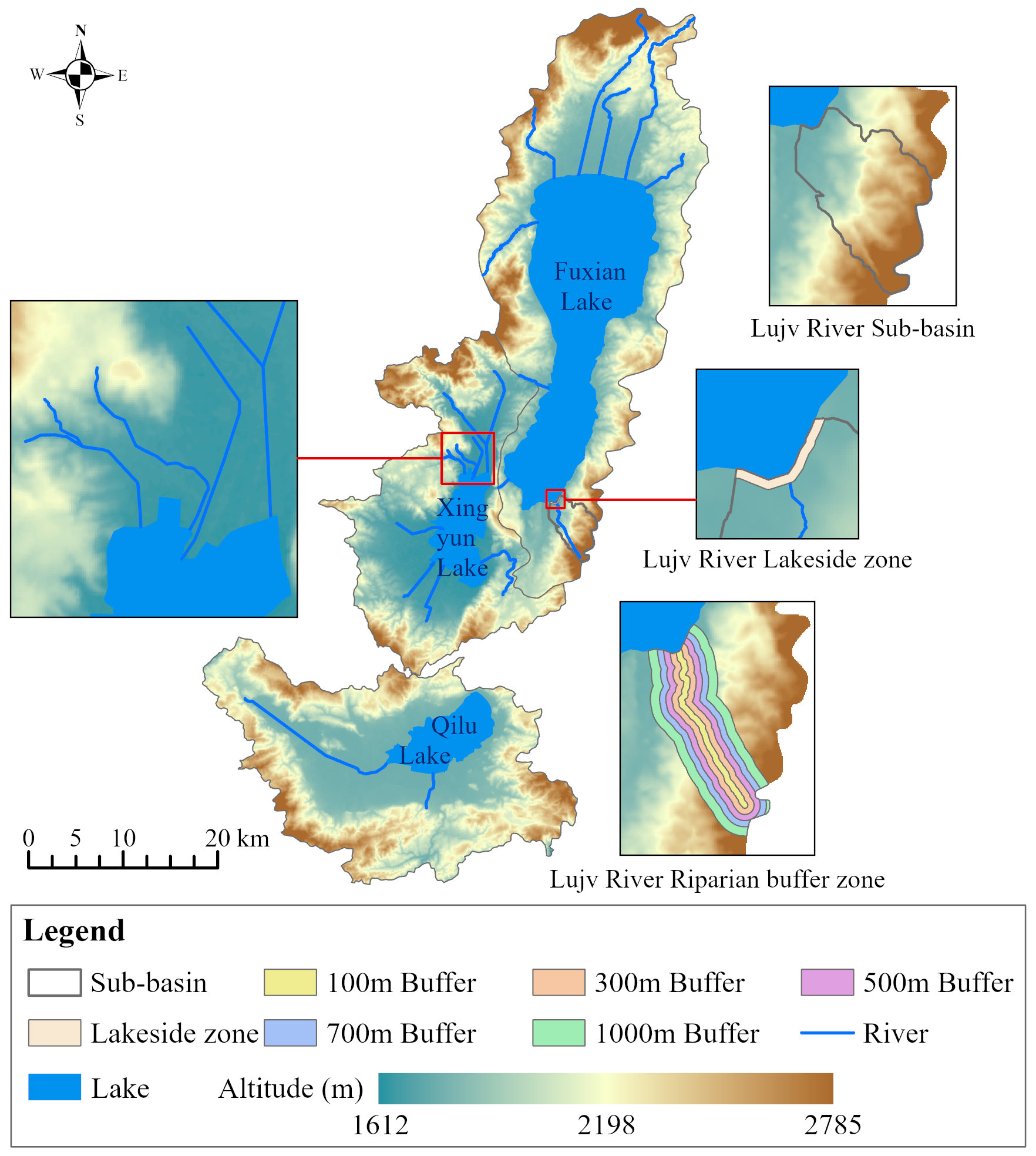

Supplement: Supplemental Information 2 [file peerj-12-18247-s002.jpg]
